# Highly sensitive piezoresistive and thermally responsive fibrous networks from the in situ growth of PEDOT on MWCNT-decorated electrospun PU fibers for pressure and temperature sensing

**DOI:** 10.1038/s41378-023-00593-1

**Published:** 2023-09-15

**Authors:** Yunyun Luo, Libo Zhao, Guoxi Luo, Linxi Dong, Yong Xia, Min Li, Ziping Li, Kaifei Wang, Ryutaro Maeda, Zhuangde Jiang

**Affiliations:** 1 State Key Laboratory for Manufacturing Systems Engineering, International Joint Laboratory for Micro/Nano Manufacturing and Measurement Technologies, Xi’an Jiaotong University (Yantai) Research Institute for Intelligent Sensing Technology and System, Xi’an Jiaotong University, Xi’an, China; 2 School of Mechanical Engineering, Xi’an Jiaotong University, Xi’an, China; 3 Shandong Laboratory of Yantai Advanced Materials and Green Manufacturing, Yantai, China; 4 Ministry of Education Engineering Research Center of Smart Microsensors and Microsystems, College of Electronics and Information, Hangzhou Dianzi University, Hangzhou, China; 5 Department of Emergency, The First Affiliated Hospital of Xi’an Jiaotong University, Xi’an, China

**Keywords:** Sensors, Nanoparticles

## Abstract

Flexible electronics have demonstrated various strategies to enhance the sensory ability for tactile perception and wearable physiological monitoring. Fibrous microstructures have attracted much interest because of their excellent mechanical properties and fabricability. Herein, a structurally robust fibrous mat was first fabricated by electrospinning, followed by a sequential process of functionalization utilizing ultrasonication treatment and in situ polymerization growth. Electrospun polyurethane (PU) microfibers were anchored with multi-walled carbon nanotubes (MWCNTs) to form conductive paths along each fiber by a scalable ultrasonic cavitation treatment in an MWCNT suspension. After, a layer of poly(3,4-ethylene dioxythiophene) (PEDOT) was grown on the surface of PU fibers decorated with MWCNTs to enhance the conductive conjunctions of MWCNTs. Due to the superior electromechanical behaviors and mechanical reinforcement of PEDOT, the PEDOT/MWCNT@PU mat-based device exhibits a wide working range (0–70 kPa), high sensitivity (1.6 kPa^−1^), and good mechanical robustness (over 18,000 cycles). The PEDOT/MWCNT@PU mat-based sensor also demonstrates a good linear response to different temperature variations because of the thermoelectricity of the PEDOT/MWCNT composite. This novel strategy for the fabrication of multifunctional fibrous mats provides a promising opportunity for future applications for high-performance wearable devices.

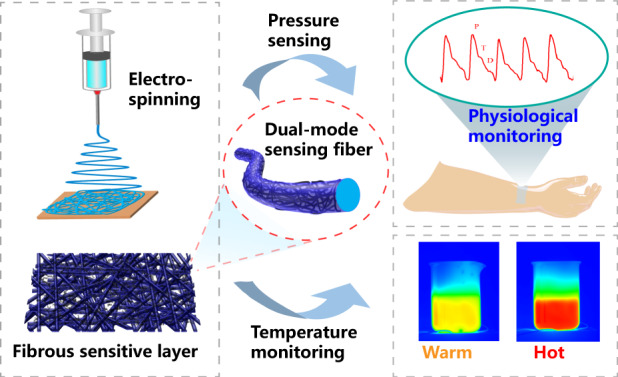

## Introduction

The growing emergence of flexible electronics has been proposed to fulfill the demands of bionic electronic skin, health monitoring, robotic sensing, and human‒machine interfaces^1–4^. To mimic the human tactile sensory ability for detecting various stimuli (pressure, strain, temperature, etc.), various piezoresistive, piezoelectrical, capacitive, or iontronic tactile sensors have been developed to encode external mechanical and thermal effects into recognizable electrical signals^5–7^. Standalone stretchable sensing platforms driven by human motion are constructed for physiological condition monitoring and body movement detection^8^. The functional circuits on a 3D freeform surface were explored to fit curvilinear skin to ensure robust performance over the shape transformation^9^. The ultrathin, high performance and skin attachable pressure sensor was used for subtle body movements^10^. The pressure sensor with ultrahigh sensitivity is capable of detecting respiration rate, respiratory depth, and respiratory arrest for early self-identification of opioid overdose^11^. A multifunctional pressure sensor was developed to capture dynamic and static stimuli^12^. Among these methods, self-powered electronic devices based on piezoelectric, electrostatic, or triboelectric mechanisms are confined to detecting dynamic signals^13–15^. Capacitive or iontronic flexible electronics are susceptible to environmental noise because of parasitic effects^16,17^. In this regard, flexible resistive tactile sensors have been extensively developed by researchers because of their high performance, simple design, convenient detection, adjustable sensitivity, and detection range^18,19^. The performance of piezoresistive sensors is related to geometric changes in the designed structure and resistivity changes in the materials^20^. Considering that most of the resistive materials are composed of conductive fillers, the sensitivity of the tactile sensor that can be achieved by adjusting its resistivity is limited^6^. Therefore, multiple methods have been adopted to fabricate various microporous structures to improve the deformation of resistive tactile sensors^21,22^. Lithography methods, natural structure reproduction, laser processing, and electrospinning technology have been widely used to fabricate controllable microstructures to achieve high sensitivity of tactile sensors^23–25^. By comparison, electrospinning is a facile, low-cost and efficient technique that has been used for the preparation of fibrous mats with consistent microporous morphology^26,27^.

To realize the functional features of the electrospun fibrous mat, several strategies of surface modification on the nanofibers have been performed that achieve a hierarchical microstructure by various coating techniques, including physically absorbed coating^28–31^, chemical polymerization coating^32^, and ultrasonication-assisted anchoring^33^. For instance, spraying, dip-coating, and filtration are generally employed strategies to adsorb functional nanomaterials on electrospun fibers through hydrogen bonding or electrostatic adherence. The spinning protein fiber was sprayed with Mxene nanosheets between the multiple fibrous layered structures for the pressure sensor exhibiting a sensitivity of 0.028 kPa^−1^ in the range of 0–40 kPa^34^. The electrospun polyurethane (PU) fibrous network was dip-coated by multiwalled carbon nanotubes (CNTs) with a pressure range of 10 kPa, a high sensitivity up to 2 kPa^−1^ and cyclic durability for 1000 repetitions^35^. The unique networks based on electrospun polyacrylonitrile (PAN) nanofibers composed of in situ growth ZIF-67 and filtration of Mxene solution resulted in a high ultrasensitive response from the rough structure, which displayed a large working range of 0–100 kPa and an ultrahigh sensitivity^36^. Nevertheless, the conductive nanomaterials adsorbed on the surface of nanofibers through surficial coating were partly detached after long-term and repeated mechanical loading, which inevitably weakened the stability and sensitivity of the fibrous sensor. Furthermore, a polymerized coating technique was developed to strengthen the fibrous structure by growing a layer of conductive polymer. Under this strategy, a graphene oxide (GO)-doped PU elastic fibrous substrate was coated with poly(3,4-ethylene dioxythiophene) (PEDOT) by solution polymerization to achieve a wider sensing range (from 14 kPa to 20 kPa), excellent cycling stability, and repeatability (over 10,000 times)^37^. The fibers uniformly covered with PEDOT via vapor phase polymerization (VPP) were utilized for blood pressure and respiratory rate monitoring for poor surface wettable materials^32^. Although the uniformly polymerized coating on the fibrous mat improved the mechanical durability of the porous structure, it was not completely satisfactory in terms of sensitivity and response time due to the enhancement of viscoelasticity. In addition, the ultrasonication anchoring technique was developed to form a conductive coating by strongly embedding nanomaterials onto the electrospun fibers. During the ultrasonic cavitation treatment, the collapse of cavitation bubbles generates a transient high temperature, microjets, and intense shock waves^38^, which could drive the nanoparticles to anchor into the electrospun fiber surface^39^. The electrospun fibers decorated with nanoparticles on the surface exhibited a higher Young’s modulus and tensile strength with excellent durability (over 10,000 cycles) for strain sensing^29,33^. The hierarchical structure from the nanoparticle decoration of the fibrous mat could be utilized to improve the performance by the formation of multiple conductive paths. On the other hand, multifunctional sensing capability is essential for practical multipurpose applications. The composite of cotton coated by reduced GO and CNTs was resistively sensitive to temperature and pressure^40^. The multifunctional sensor cannot effectively discriminate between temperature and pressure stimuli via variations in resistance, despite that unique recognition of external stimuli is highly desirable. Although the skeleton of the electrospun fibrous structure shows a high sensitivity to external pressure, it also reduces the pressure range and increases the response time of the devices due to the hysteresis of the porous structure. Such single-step surface modifying techniques have limited effects on the mechanical properties of the fibrous composite materials. It thus remains an essential challenge to realize the mechanical robustness, stability, multifunctionality, and ultrahigh sensitivity of the designed tactile sensor^41–43^.

Here, we report a novel strategy of ultrasonication anchoring followed by in situ polymerization for preparing a highly sensitive PEDOT/MWCNT@PU electrospun fibrous mat. Thermoplastic PU fibrous mat was electrospun as the skeleton of the porous structure. Then, the electrospun fibrous PU mats were immersed into an MWCNT suspension and treated by high-power ultrasonication to anchor MWCNTs onto the surface of the electrospun PU fibers. The junctions of MWCNTs were covered and embedded onto PU fibers to form conductive paths. After, PEDOT was polymerized through the VPP technique on the skeleton of the hierarchical fibrous structure to fabricate the PEDOT/MWCNT@PU composite fiber. The PEDOT/MWCNT@PU mat was highly sensitive to external mechanical stimuli, showing a high sensitivity of 1.6 kPa^−1^ over a wide sensing range (0–70 kPa). Furthermore, the fibrous mat-based sensor showed excellent biological signal monitoring capability and outstanding durability. In addition, the thermoelectric characteristics of the PEDOT/MWCNT components enable the fibrous mat to be used for temperature sensing. The temperature response exhibited good linearity, and the sensor was applied to measure the temperature of objects in contact. Based on its favorable performance, this proposed fibrous mat-based sensor is promising for future applications in physiological signal monitoring, environmental sensing, and tactile electronic devices.

## Experimental section

### Materials

Thermoplastic PU (Elastollan 1185A) was purchased from BASF Company, Germany. The MWCNTs were purchased from XFNANO Materials Tech Co., Ltd., China. Sodium dodecyl benzene sulfonate (SDBS, 99.5%, AR) and N,N-dimethylformamide (DMF, 99.5%, AR) were purchased from Sigma Aldrich, United States. Ethanol (99.5%, AR), acetone (99.5%, AR), isopropyl alcohol (IPA, 99.5%, AR), ferric chloride (FeCl_3_, 98%) and ethyl dioxythiophene (EDOT, 99%) were purchased from Aladdin Biochemical Technology Co., LTD, China. PU adhesive was purchased from Hongsheng Medical Technology Co., LTD, China. The interdigital electrodes were commercially customized by Shenya Precision Circuit Company.

### Preparation of the PEDOT/MWCNT@PU mat

The PU pellets (30%, w/v) were dissolved into a mixture of DMF/acetone (volume ratio, 1:1) under magnetic stirring for 6 h to form a homogeneous precursor solution. The PU solution was electrospun at 15 kV, associated with a feed rate of 1 mL/min, with a distance of ca. 15 cm between the high voltage applied nozzle and collector board. The thickness of the PU mat can be adjusted by the spinning time from 100 μm to 400 μm. The as-prepared PU fibrous mat was peeled off and dried in an oven at 60 °C for 12 h to evaporate the residual solvent. After that, 50 mg of MWCNTs and 25 mg of SDBS were dispersed into 100 mL of deionized (DI) water under ultrasonication treatment for 30 min to obtain a homogeneous MWCNT suspension. The tailored PU mats were immersed into MWCNT dispersions and treated by an ultrasonic horn with a power of 400 W for 1 h (JY98-IID, Ningbo Scientz Biotechnology Co., Ltd, China). The ultrasonic frequency is nominal at 20 kHz, and the dispersion is placed in an ice bath to maintain a constant temperature. Then, the MWCNT@PU fibrous mats were washed several times and dried in an oven at 60 °C for 12 h. Next, the MWCNT@PU mats were immersed in a 10 wt% FeCl_3_ solution (IPA) for 5 min and dried in an oven at 50 °C. The MWCNT@PU(FeCl_3_) mat was suspended inside a Schlenk flask, and sufficient EDOT was placed in the bottom of the flask. The vapor VPP process was carried out at 60 °C for 12 h under vacuum conditions. After polymerization, the PEDOT/MWCNT@PU mat was rinsed with ethanol and DI water continuously and dried in an oven at 50 °C.

### Fabrication of the flexible pressure sensor and temperature sensor

A piece of as-prepared PEDOT/MWCNT@PU mat (8 mm × 10 mm) was attached to a customized interdigital electrode for pressure sensing. The PU medical dressing was used to bind the PEDOT/MWCNT@PU mat and interdigital electrode. The 4 × 4 pressure sensing array adopted the same method for encapsulation to enhance the device’s robustness. Both ends of a rectangular PEDOT/MWCNT@PU mat (4 mm × 10 mm) were connected to the copper electrode with conductive silver paste for temperature sensing.

### Characterization and performance test

The morphologies and microstructures of the PEDOT/MWCNT@PU fibers were observed by scanning electron microscopy (SEM, Hitachi SU8010, Japan), including energy dispersive spectrometry (EDS) mapping. Transmission electron microscopy (TEM, JEM-F200, JEOL, Japan) was adopted for characterization of the decoration of MWCNTs and the polymerization of EDOT. Fourier transform infrared (FTIR) spectroscopy (Bruker VERTEX70, USA) was performed to observe the feature bands of the composite fibers. Raman spectroscopy was performed using a laser Raman spectrometer (InVia Qontor, Renishaw) with a 532 nm laser for excitation. Thermogravimetric analysis (TGA) was performed by differential thermal analysis (Pyris 1 TGA, PerkinElmer). Compression tests were carried out by a Universal Material Testing Machine (Zhuhai SUST electrical equipment Co, LTD, China). The tested mat was cut into a size of 20 mm × 20 mm, which is the same as the size of the customized indenter. The compression processes were set at a strain rate of 0.05 mm/min and a prestress of 5 mN. Electrical measurements were taken with a digit precision multimeter (DMM 6510, Keithley). I–V curves of fabricated pressure sensors based on the PEDOT/MWCNT@PU mat were tested by an electrochemical station (Reference 600 + , Gamry). A four-probe resistivity tester (HPS2662, HELPASS, China) was used for resistivity measurement. The humidity was set by a humidity controller (SHSH-D6000, China). A thermal infrared imager (X6520sc, Switzerland) was adopted for the measurement of the temperature gradient.

## Results and discussion

**Fig. 1:**
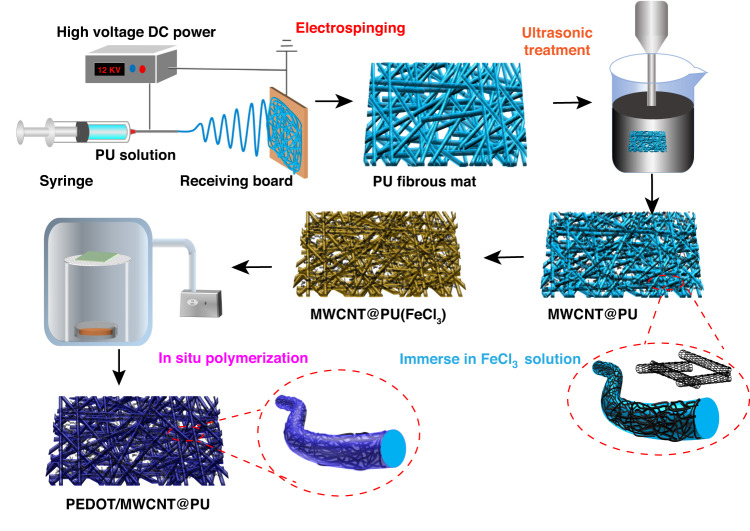
Preparation of the sensitive fibrous layer. Schematic of the PEDOT/MWCNT@PU mat preparation

**Fig. 2:**
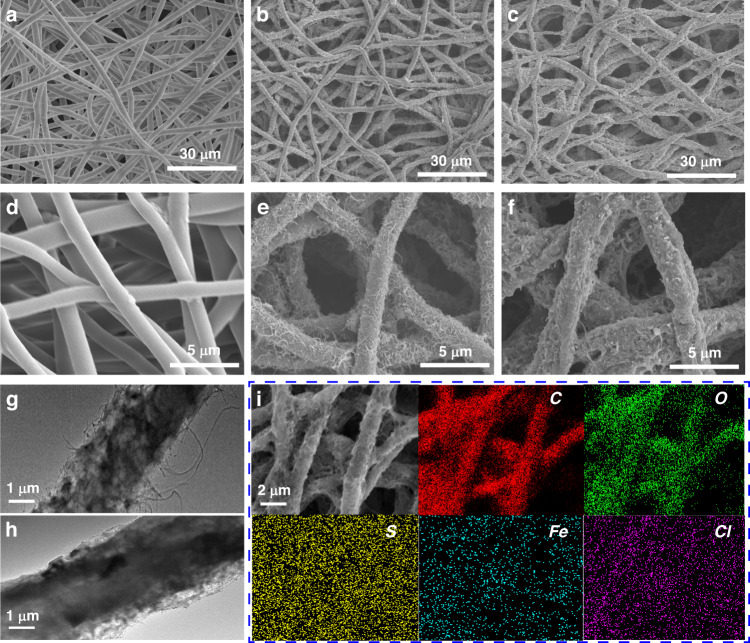
Microstructure of the as-prepared fibers. **a** PU fibers. **b** MWCNT@PU fibers and **c** PEDOT/WMCNT@PU fibers. **d**–**f** High-magnification SEM images of **a**–**c**. **g**–**h** TEM images of WMCNT@PU and PEDOT/WMCNT@PU. **i** EDS mapping of PEDOT/WMCNT@PU

Figure 1 shows the schematic process for PEDOT/MWCNT@PU mat preparation. The PU fibrous mat was prepared by electrospinning. The MWCNT@PU mat was obtained through ultrasonic treatment in MWCNT dispersion solution through the strong impact generated from the collapse of cavitation bubbles during the ultrasonic cavitation process^39^. As the MWCNTs were decorated onto the surface of PU fibers, the fibrous mat changed from white (Fig. S1a) to black (Fig. S1b). After immersion in FeCl_3_-IPA solution, the EDOT monomer is polymerized in situ on the surface of MWCNT@PU (FeCl_3_) fibers by VPP. The PEDOT/MWCNT@PU mat was obtained after facile polymerization (Fig. S1c). As shown in Fig. 2a, d, the

The pristine PU fibers show a relatively smooth surface. These PU fibers were fully anchored and covered with MWCNTs after 1 h of ultrasonication treatment. The surface of MWCNT@PU fibers exhibited a hierarchical structure for the mutual wrapping and conjunction of MWCNTs, as shown in Fig. 2b, e. SEM inspection (Fig. 2c, f) revealed the irregular roughness of PEDOT/MWCNT@PU fibers after the in situ polymerization of PEDOT on the surface of MWCNT@PU fibers. In addition, TEM inspection (Fig. 2g) of an MWCNT@PU fiber clearly indicated that the MWCNTs were embedded into the PU fiber and interlocked with one another to form conductive paths. Figure 2h shows the core-shell structure of the PEDOT/MWCNT@PU fiber, and the shell was composed of a layer of PEDOT/MWCNT composite, in which PEDOT wrapped the MWCNT network. Experimentally, the electrical surface resistivities of the MWCNT@PU and PEDOT/MWCNT@PU fiber mats are 316.5 Ω·cm and 105.8 Ω·cm, respectively. The conductivity of the sample was greatly improved after the polymerization of PEDOT. EDS mappings (Fig. 2i) show the distribution of the C, O, S, Fe, and Cl elements. The uniform distribution of S as the featured element confirms the existence of PEDOT.

**Fig. 3:**
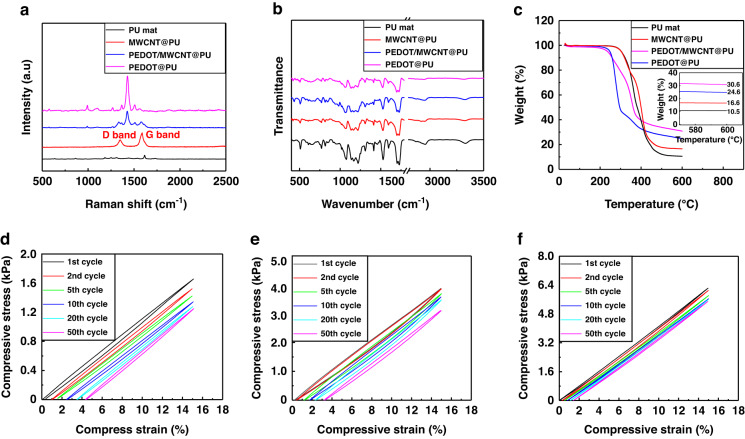
Characterization and compressive mechanical properties of the prepared fibrous mat. **a** Raman spectra. **b** FTIR spectroscopy. **c** TGA analysis. **d**–**f** Compressive mechanical properties of the PU mat. MWCNT@PU mat. PEDOT/MWCNT@PU mat

Raman spectra were recorded to confirm the characteristic bands of MWCNTs and PEDOT. As shown in Fig. 3a, the characteristic bands of carbon materials appear at 1352 cm^−1^ (D band) and 1593 cm^−1^ (G band) for the MWCNT@PU fibers. PEDOT was characterized by bands at 1434 cm^−1^ and 1502 cm^−1^ for the C-C interring and C = C stretching^44^. Notably, the bands of the PEDOT/MWCNT@PU mat shift to 1430 cm^−1^ and 1505 cm^−1^ as a result of the interfacial interaction of MWCNTs and PEDOT. In addition, FTIR results (Fig. 3b) were introduced to analyze the vibration bands of functional groups. The characteristic bands of PU at 1072 cm^−1^ and 1595 cm^−1^ are consistent with the vibrations of C-O and N-H, and the vibrations at 2955 cm^−1^ and 3332 cm^−1^ correspond to the -CH_3_ group and overlapping stretching of N-H^45^. The unique characteristic band of PEDOT at 980 cm^−1^ is attributed to the vibration of C-S-C. Elemental analysis of the PEDOT/MWCNT@PU composite was recorded using TGA, as shown in Fig. 3c, based on the thermal degradation component ratio^39^. According to the weight ratios of the residuals of the as-prepared mats, the weight of MWCNTs decorated onto the PU fibers is calculated as 6.8%. According to the mass fraction of MWCNTs, the conductivity of the MWCNT@PU mat satisfies the classical percolation model^46^. The in situ growth of PEDOT accounts for 16.8% of the composite fiber mat in weight. The conductive paths are greatly increased by the adhesion of PEDOT, which is beneficial to the improvement of electrical properties.

Furthermore, the mechanical properties of the fibrous mats were tested by using cyclic compression experiments. The PU fibrous mat, MWCNT@PU, and PEDOT/MWCNT@PU mats were fabricated into the same size of 20 mm × 20 mm with a thickness of ca. 300 μm. As shown in Fig. 3d–f, there were residual strains of 4.4%, 3.3%, and 1.4% from the PU, MWCNT@PU, and PEDOT/WMCNT@PU mats, respectively, after 50 cycles with the same compression strain of 15%. PEDOT/WMCNT@PU showed a maximum compressive stress of 6.3 kPa at a strain of 15%, which is higher than those of 3.8 kPa (MWCNT@PU) and 1.7 kPa (PU). In addition, the PEDOT/WMCNT@PU mat maintained 87% of its maximum compressive stress after 50 cycles at a strain of 15% compared with 80% of MWCNT@PU and 75% of the PU mat. These residual strains of tested mats are generated from the viscoelasticity of polymers^47^, which affects the mechanical robustness of the fabricated sensor. As the MWCNTs were decorated onto the PU fibers, the elastic modulus of the fibrous mat was increased due to the wrapped WMCNTs acting as reinforcing ribs that strengthen the compressive mechanics. As indicated by the compression experiment, the PEDOT/MWCNT@PU mat has the best compressive mechanical properties among the prepared mats due to the filling and coating of the MWCNT network by PEDOT. The composite fibers are further strengthened for tighter adhesion of MWCNTs by the PEDOT coating. The strengthening effects are suitable to cement the mechanical performance of the designed sensors, such as a wide measurement range, fast response, and mechanical robustness.

**Fig. 4:**
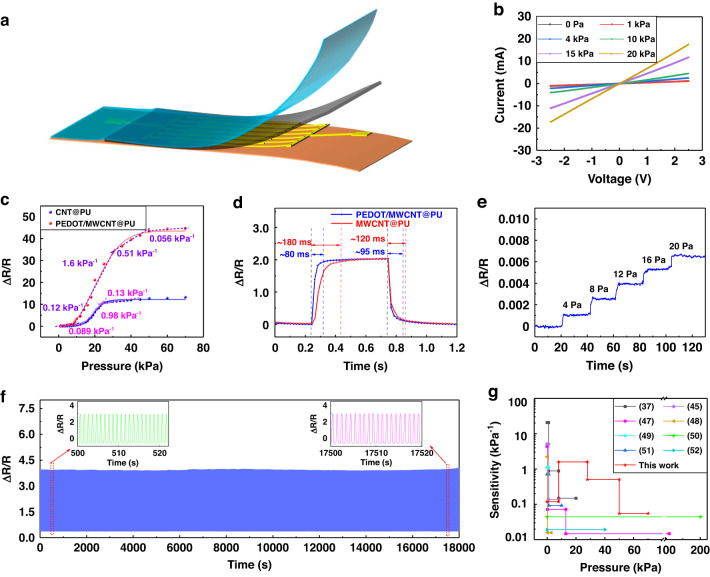
Mechanical response of the fibrous mat-based pressure sensor. **a** Illustration of the structural fabrication of the sensor. **b** Current–voltage curves. **c** Variation in the resistance and sensitivity of the PEDOT/MWCNT@PU- and MWCNT@PU-based pressure sensors. **d** The response and recovery time of the PEDOT/MWCNT@PU and MWCNT@PU-based pressure sensors. **e** Continuous low-pressure loading detection. **f** Long-term loading/unloading cycles for stability. **g** Comparison of the performance of fibrous pressure sensors

A schematic of the PEDOT/MWCNT@PU-based pressure sensor is shown in Fig. 4a. The pressure sensor was encapsulated with PU tape, and the PEDOT/MWCNT@PU was squeezed on the electrode using the adhesive tape. The tightly attached adhesive tape on the substrate pulled the top surface of the sensitive layer so that it had close contact with the electrodes, as shown in Fig. S2. Figure 4b shows the current-voltage curves of the pressure sensor based on the PEDOT/MWCNT@PU mat under different pressure loads, which exhibit good linearity, indicating that the sensor follows Ohm’s law. The pressure response of the prepared sensor is defined as ΔR/R_0_, where R_0_ is the initial resistance and ΔR is the variation in resistance under a specific pressure. The sensitivity is defined as (ΔR/R_0_)/ΔP. Figure 4c demonstrates the response and sensitivity of the fibrous sensor. The sensitivity of the device based on the MWCNT@PU mat is 0.089 kPa^−1^, 0.98 kPa^−1^, and 0.13 kPa^−1^ at different detection ranges of 0–12 kPa, 12–20 kPa, and 20–40 kPa, respectively. The PEDOT/MWCNT@PU sensor exhibits four ranges of linear relationships, which correspond to stage I (0–8 kPa), stage II (8–28 kPa), stage III (28–50 kPa), and stage IV (50–70 kPa), with sensitivities of 0.12 kPa^−1^, 1.6 kPa^−1^, 0.51 kPa^−1,^ and 0.056 kPa^−1^, respectively. The lower sensitivity of the sensor at low pressure is partially attributed to the prepressure generated by the adhesive tape for encapsulation. The derived pressure resulted in a small predeformation in the sensitive layer, which lowers the sensitivity at low pressure. Compared with the pressure sensor based on the MWCNT@PU mat, the PEDOT/WMCNT@PU mat-based pressure sensor has better performance not only because of a higher sensitivity but also because of a wider detection range. This can be attributed to the reinforcement of MWNT@PU by the assembly of PEDOT. The compression tests suggest that the PEDOT/MWCNT@PU mat exhibits a higher modulus of compressibility, which results in a superior working range and faster response ability than other composites. The response/recovery time was 80/95 ms for the PEDOT/MWCNT@PU-based sensor (Fig. 4d). In contrast, the MWCNT@PU-based device showed a larger response time of ~180 ms and a recovery time of 120 ms for the viscoelasticity of the composite material. Figure 4e shows that the fibrous sensor has an ultralow detection limit. With this response, it is easy to distinguish 0–20 Pa by a continuous step loading of a 4 Pa pressure. Figure 4f shows the stability of the fabricated sensor for 18,000 loading cycles, where the response of the sensor remains almost identical under the same pressure. This excellent stability performance is attributed to the reinforced mechanical properties of the PEDOT/MWCNT@PU mat and the prominent encapsulation. In contrast, the performances of various pressure sensors from previously reported works are listed in Fig. 4g and Table S1^37,45,47–52^. Compared with these works, our sensor demonstrates superior sensitivity, limit of detection, and stability across a wide working range.

**Fig. 5:**
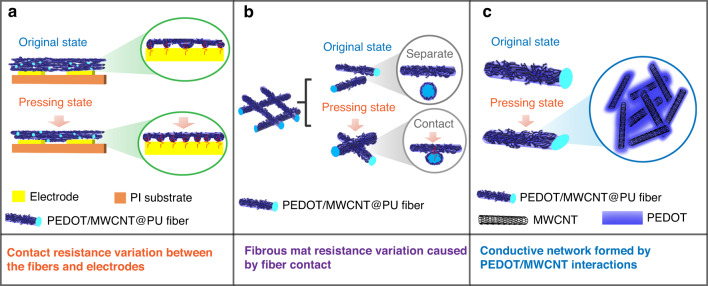
Schematic illustration of the pressure sensing mechanisms of the sensor based on the PEDOT/MWCNT@PU mat. **a** Contact resistance variation between the fibers and electrodes. **b** Fibrous mat resistance variation caused by fiber contact. **c** Conductive network by PEDOT/MWCNT interactions

The pressure-sensitive mechanisms are related to the contact resistance between the fibrous mat and the electrodes, the bulk fibrous mat resistance variation caused by the fiber contact, and interactions between the MWCNTs and PEDOT sheets. As Fig. 5a shows, the pressed contact points between the PEDOT/MWCNT@PU mat and electrodes increase with the application of pressure, and the contact resistance decreases with increasing pressure. In addition, the contact area and contact points between the fibers both increase under the pressed state because the separate fibers come into close contact, as shown in Fig. 5b. On the smaller microscale networks, the conductive network of the PEDOT/MWCNT decorated on the PU fibers would change the inter-MWCNT distance during the loading process. The adjacent PEDOT/MWCNTs would reconnect to change the formed conductive paths and cause the resistance variation, as shown in Fig. 5c.

**Fig. 6:**
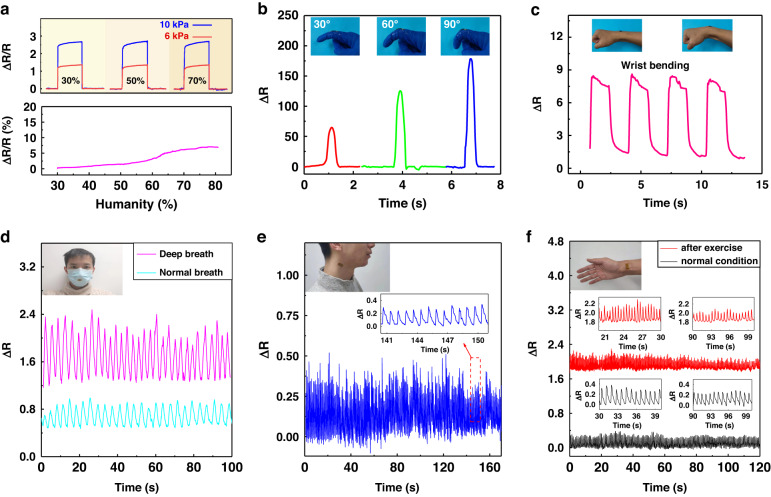
Physiological monitoring of the prepared sensor. **a** The performance of the sensor under different humidities. **b** Detection of finger bending angles on the knuckle. **c** Detection of wrist bending. **d** Respiratory monitoring under deep and normal breathing. **e** The carotid pulse monitoring. **f** Radial artery pulse monitoring after exercise and under a normal relaxed state

The outstanding perceptual performance of this pressure sensor based on the PEDOT/WMCNT@PU mat demonstrates great potential for practical applications such as human physiological signal monitoring. In the first scenario further testing this application space, the performance of the sensor in an adjustable humidity environment (30–80% RH) was recorded. The relative resistance changes caused a maximum resistance deviation of 6.8%, which was attributed to the encapsulation of the PU dressing tape insulating the adsorption of water moisture. The performance of the sensor is minimally affected by the relative humidity (Fig. 6a). Thus, the sensor could be directly attached to the skin without risk of issues caused by water moisture and sweating on the sensor. The sensor can therefore be used to detect the bending situation caused by human movement, due to the resistance change of the sensor being predominantly caused by pressure changes^53^. The strain generated by different bending angles can be calculated, and the response is recorded in Fig. S3. The sensor can decouple the bending strain and pressure when they are simultaneously applied^54^. The positive/negative strain in the upper/lower neutral layer during bending of the sensitive layer weakens the bending effect (Fig. S4). The different finger bending angles and wrist bending are monitored in Fig. 6b, c. To validate the capability of monitoring subtle motions, the sensor was attached to the outside surface of a disposable surgical mask to record the respiratory process. The small pressure induced by airflow from breathing was tested under different states of deep breathing and normal breathing, as shown in Fig. 6d. The deep breath process was taken at a frequency of 19 times per minute with a larger output, which was clearly distinguished from the weaker response of the slower normal breath at 17 times per minute. In addition, the sensor was tested while attached to the neck to detect the carotid pulse. The carotid pulse rate measured by the sensor was 81 beats per minute, as shown in Fig. 6e, which was in good agreement with the heart rate test on a commercial Mi Band 6 (Fig. S5). Furthermore, the sensor was fixed on the wrist to check the variation in the radial artery pulse under different movement statuses, as shown in Fig. 6f. The radial pulse in a normal relaxed state for two minutes remained steady and retained a constant pulse rate of 81 times per minute. After the participant wore the sensor and engaged in a fast and strenuous five-minute run, the pulse rate gradually dropped from 135 to 117 times per minute, and the pulse force slowed down in the following two minutes. The enlarged pulse waves clearly show the detailed percussion wave (P-wave), tidal wave (T-wave), and diastolic wave (D-wave) in Fig. S6. The data show that the T and D waves of the radial pulse after exercise are less regular and exhibit steeper slopes. High-quality pulse waveforms are vital for the feature extraction of the time domain, frequency domain, and morphological characteristics of valid signals. These continuous high-precision feature extractions have great potential in pulse waveform analysis for cardiovascular health monitoring by data analysis algorithms. Through comparative analyses, the valuable information of the monitored pulse waves could be used to prediagnose the physical state.

**Fig. 7:**
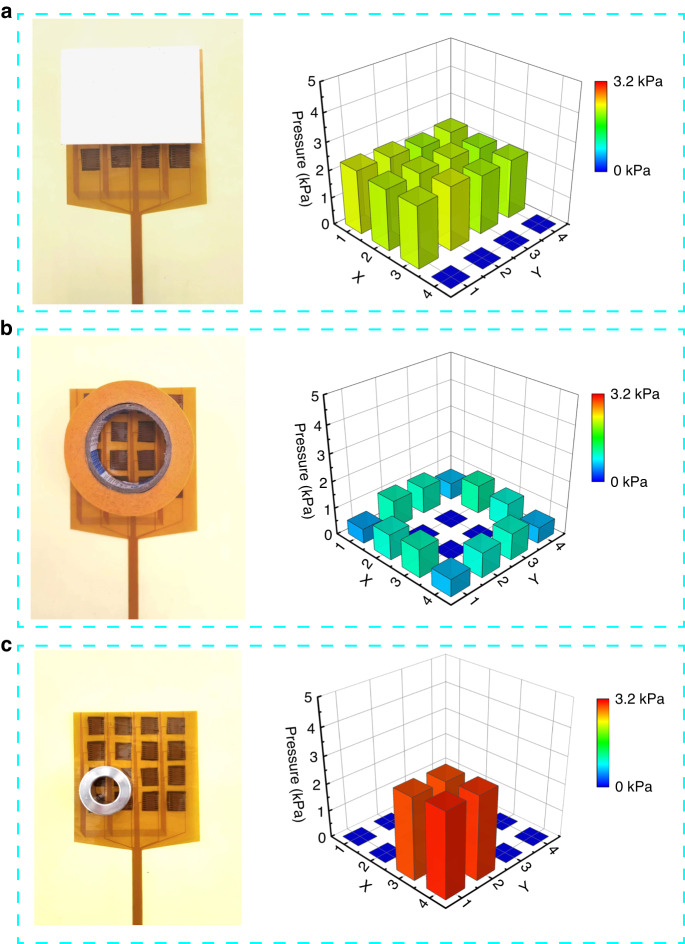
Force mapping of the pressure sensor array. **a** Plastic cuboid. **b** Paper adhesive tape. **c** Stainless steel ring

Furthermore, a monolithic 4 × 4 sensor array with a size of 8 mm × 8 mm per pixel was fabricated for force mapping. As shown in Fig. 7, three objects with weights of 148 g, 38 g, and 52 g were placed on the array, and the visualized pressure mappings were exhibited with the pressure distribution for different pressures and shapes. A plastic cuboid shows a rectangular pressure distribution in the range of 2.1–2.3 kPa (Fig. 7a). A roll of paper adhesive tape presents a circular and small pressure distribution due to its low mass density, as shown in Fig. 7b. A stainless steel ring was mapped at the left bottom in a range of 3.1–3.2 kPa pressure distribution (Fig. 7c). The varied position of the object placed on the sensor array was monitored (Movie S1). In this demonstration, the sensor array shows potential applicability for detecting the shape and weight of different objects in human‒machine interactions.

**Fig. 8:**
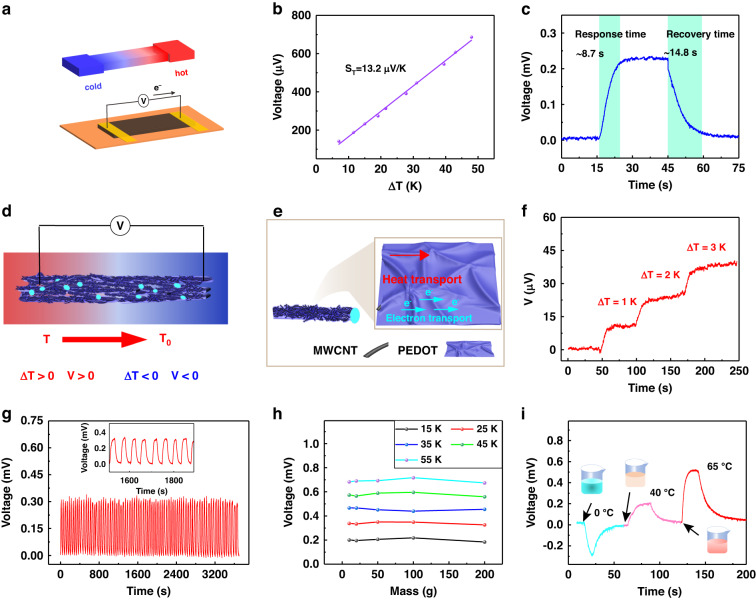
Thermal electricity of the PEDOT/MWCNT@PU mat. **a** Structural schematic illustration of PEDOT/MWCNT@PU for temperature sensing. **b** Relationship between the voltage output and temperature gradient. **c** Response and recovery time for temperature sensing. **d** The working mechanism of the PEDOT/MWCNT@PU mat. **e** Mechanisms of the thermoelectric performance of PEDOT/MWCNTs. **f** Output voltage with a low temperature difference. **g** Voltage output for 80 cycles at a temperature gradient. **h** Voltage variation against various weights at different temperatures. **i** Response to the different water temperatures

In addition, the thermal response of the PEDOT/MWCNT@PU mat was evaluated considering the good thermoelectric properties of the PEDOT/MWCNT composite. Figure 8a shows the structural schematic illustration of the PEDOT/MWCNT@PU-based temperature sensor. The thermoelectric voltage is generated by the Seebeck effect through the PEDOT/MWCNT@PU fibrous mat, as shown in Fig. 8d, e. When the temperature gradient is applied across the composite fibers, the heat transfer causes charge carriers to transport through the PEDOT/MWCNT composite^55^. The voltage output (V) is linear with a temperature gradient (ΔT), and the Seebeck coefficient (S_T_) of the fabricated thermoelectric sensor can be calculated as S_T_ = V/ΔT. A semiconductor heating device was used to calibrate the temperature gradients, and the linear relation between the outputs and the temperature gradients was obtained, as shown in Fig. 8b. In the temperature gradient from 7 to 48 K, the sensitivity of the sensor is 13.2 μV/K, and the thermoelectric voltage response is demonstrated in Fig. S7. Due to the low thermal conductivity of the sensitive layer caused by the fibrous structure, the response time of the prepared sensor is 8.7 s, and the recovery time is 14.8 s (Fig. 8c). A low temperature gradient of 1 K was achieved, as shown in Fig. 8f. Moreover, the sensor exhibited high cycling stability at 0 K and 23 K for 80 cycles, as shown in Fig. 8g. Considering the sensitive layer with two different sensitive mechanisms, piezoresistive and thermoelectric, the effect of external pressure on the temperature sensor was also investigated. As Fig. 8h shows, the outputs of the temperature sensor were slightly affected by the application of different weights above the sensitive layer, confirming the independence of temperature sensing and mechanical force sensing as achieved by the two different sensing mechanisms^55^. Finally, the fabricated sensor was used to detect cold, warm, and hot water, as shown in Fig. 8i. Along with the increasing temperature gradients, the outputs of the sensor also increased linearly. These results validate that the PEDOT/WMCNT@PU mat has potential applications for the perception of environmental temperature.

**Fig. 9:**
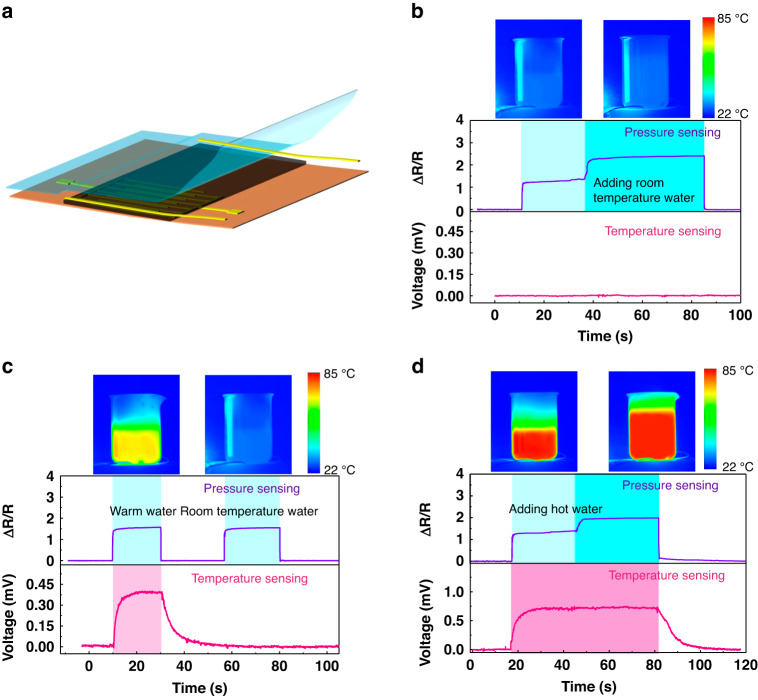
Applications of the prepared sensor for pressure and temperature sensing. **a** The schematic diagram for pressure and temperature sensing, **b** pressure sensing without temperature applied, **c** the same pressure with different temperatures, and **d** the same temperature with different pressures

A device was prepared for pressure and temperature sensing by stacking the electrodes on the two sides of the PEDOT/WMCNT@PU mat, as shown in Fig. 9a. The pressure and temperature stimuli were measured by detecting the resistance and voltage variations. To simultaneously verify the capability of monitoring pressure and temperature stimuli, we designed several applications in which temperature and pressure were applied to evaluate the performance of this device. First, when a beaker filled with half and a cup of room temperature (25 °C) water was placed on the sensor, the pressure-sensitive relative resistance was varied from 1.3 to 2.4. The voltage output of the sensor remained unaffected because there was no temperature gradient, as shown in Fig. 9b. However, when warm water (55 °C) was utilized, as shown in Fig. 9c, a clear temperature response (0.4 mV) was observed through the voltage signal by the thermoelectricity. In contrast, a beaker filled with an equal amount of room temperature water did not generate a thermoelectric voltage, and the pressure response remained the same without any interference from temperature, verifying that the pressure sensitivity is not affected by the temperature, as shown in Fig. 9c. Furthermore, as shown in Fig. 9d, when a beaker filled with hot water (78 °C) was added, the output voltage remained constant, and the resistance of the pressure sensor varied with the volume fluctuation of hot water, illustrating that the temperature response was not affected by the pressure (Fig. 9d). These results demonstrate independence of pressure and temperature sensing using the fibrous PEDOT/WMCNT@PU sensor. Compared with the pressure and temperature sensors in reported studies (Table S2), the fibrous PEDOT/WMCNT@PU sensor exhibits high sensitivity, low detection limit, rapid response/recovery speed, and long-term stability and repeatability, which supports its potential capability for serving diverse application scenarios.

## Conclusion

In conclusion, we have presented a strategy for the fabrication of a highly sensitive piezoresistive and thermally responsive fibrous mat. Toward overcoming the softness of electrospun fiber mats, an ultrasonication-assisted anchoring technique and an in situ polymerization process were adopted to reinforce the mechanical properties of electrospun PU mats by embedding MWCNTs and wrapping them with PEDOT shells, respectively. The PEDOT/MWCNT@PU mat exhibited an excellent resilience ability, maximum compressive stress and minor residual strains through 50 compression experiments, which also highlighted its stability and durability through cyclic testing. In addition, the PEDOT/MWCNT@PU fibrous networks present novel properties, such as a high sensitivity, fast response, excellent cycling stability, and low detection limit for pressure sensing. Physiological signals indicating respiratory, carotid, and radial artery pulse activity were continuously monitored, and an array of pressure sensors was used to map the pressure distribution of the applied objects. Furthermore, the thermoelectricity of the fibrous composite also demonstrated the ability for temperature sensing. Due to its excellent properties, the PEDOT/WMCNT@PU fibrous mat with temperature and pressure sensing ability presents widely promising application prospects in wearable health care monitoring and electronic skin.

### Supplementary information

Highly sensitive piezoresistive and thermal responsive fibrous networks from in-situ growth PEDOT on MWCNT decorated electrospun PU fibers for pressure and temperature sensing

Movie S1
