# Supplementary material for: Highly sensitive piezoresistive and thermally responsive fibrous networks from the in situ growth of PEDOT on MWCNT-decorated electrospun PU fibers for pressure and temperature sensing
Source: Microsyst Nanoeng. 2023 Sep 15;9:113. doi: 10.1038/s41378-023-00593-1 (PMC10504313; doi:10.1038/s41378-023-00593-1)
Supplement: Supplementary file 1 — Highly sensitive piezoresistive and thermal responsive fibrous networks from in-situ growth PEDOT on MWCNT decorated electrospun PU fibers for pressure and temperature sensing [file 41378_2023_593_MOESM1_ESM.docx]

**Highly sensitive piezoresistive and thermal responsive fibrous networks from in-situ growth PEDOT on MWCNT decorated electrospun PU fibers for** **pressure and temperature sensing**

Yunyun Luo^1, 2, 3^, Libo Zhao^1, 2, 3^, Guoxi Luo^1, 2, 3^*, Linxi Dong^4^, Yong Xia^1, 2, 3^*, Min Li^1, 2, 3^, Ziping Li^1, 2^, Kaifei Wang^5^*, Maeda Ryutaro^1, 2^, Zhuangde Jiang^1, 2^

^1^ State Key Laboratory for Manufacturing Systems Engineering, International Joint Laboratory for Micro/Nano Manufacturing and Measurement Technologies, Xi’an Jiaotong University (Yantai) Research Institute for Intelligent Sensing Technology and System, Xi'an Jiaotong University, Xi'an 710049, China

^2^ School of Mechanical Engineering, Xi'an Jiaotong University, Xi'an 710049, China

^3^ Shandong Laboratory of Yantai Advanced Materials and Green Manufacturing, Yantai 265503, China

^4^ Ministry of Education Engineering Research Center of Smart Microsensors and Microsystems, College of Electronics and Information, Hangzhou Dianzi University, Hangzhou 310018, China

^5^ Department of Emergency, The First Affiliated Hospital of Xi’an Jiaotong University, Xi’an 710061, China

Corresponding Author E-mail: luoguoxi@mail.xjtu.edu.cn; yongxia@xjtu.edu.cn; aifeiw@xjtufh.edu.cn.


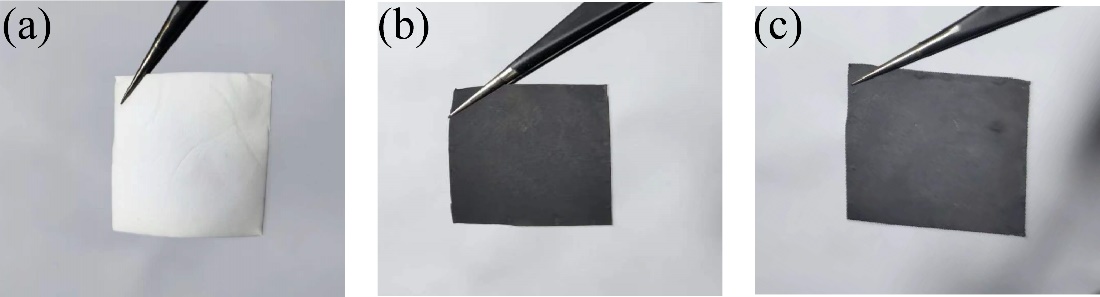


**Figure S1**. The photograph of prepared mats. (a) PU mat, (b) MWCNT@PU mat (c) PEDOT/WMCNT@PU mat.


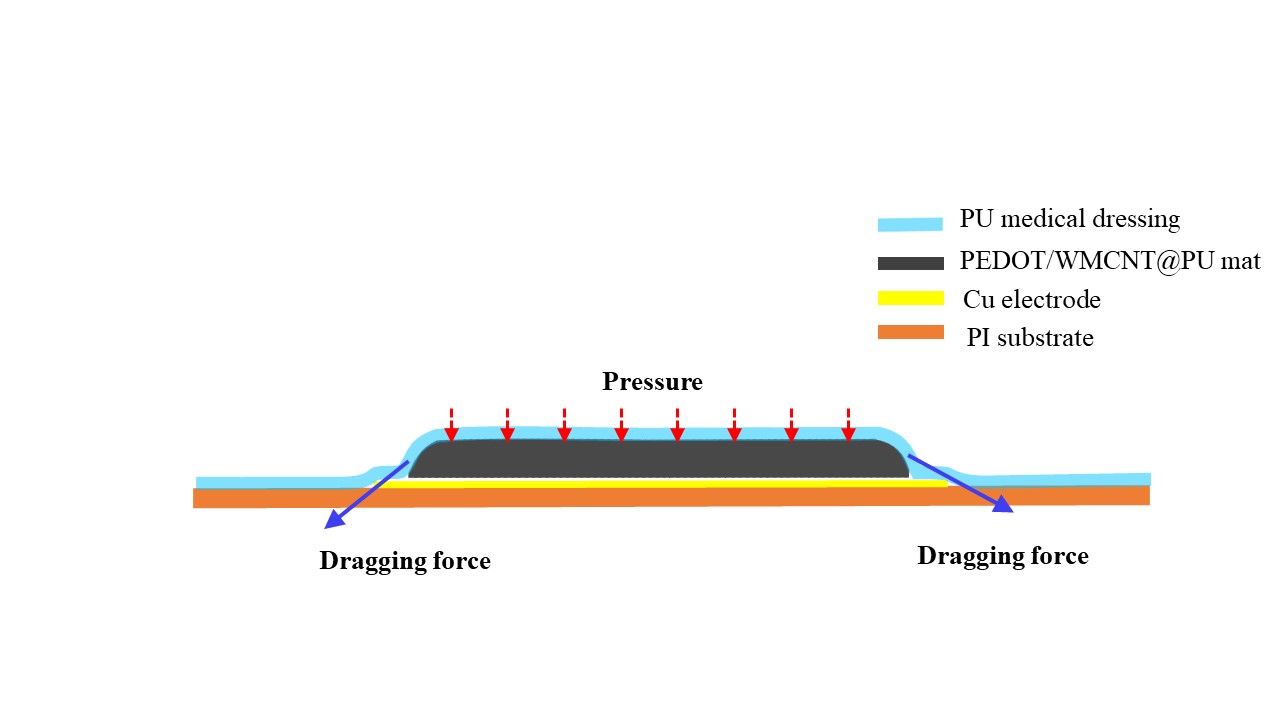


**Figure S2**. The schematic of pre-pressure generated from the PU dressing.


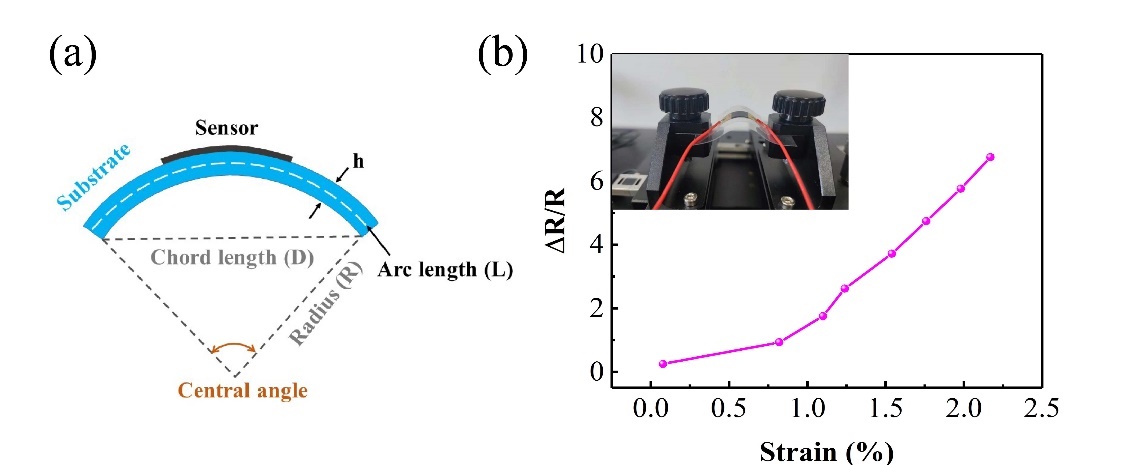


**Figure S3.** Schematic illustration of the strain testing and calculation. (a) illustration of the strain calculation, (b) Strain response of the sensor

In order to quantitatively analysis of the strain response, the sensor was bent on a 0.1mm PET film, the strain can be calculated as the expression:

L=2R×sin(D/2R) and strain ε=h/2R

where L and D represent the arc length and chord of the PET film, respectively. h is 0.1mm and a 34 mm PET film is tailored to attach the sensor.


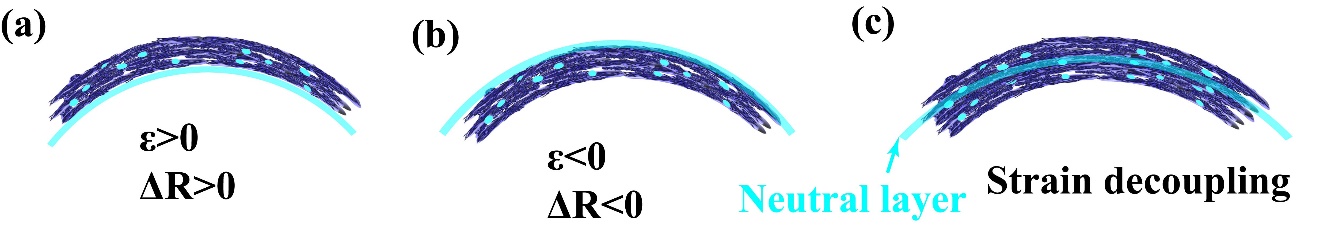


**Figure S4.** Schematic diagram of the sensor with different strain. (a) ε>0, (b) ε<0, (c) decoupling.


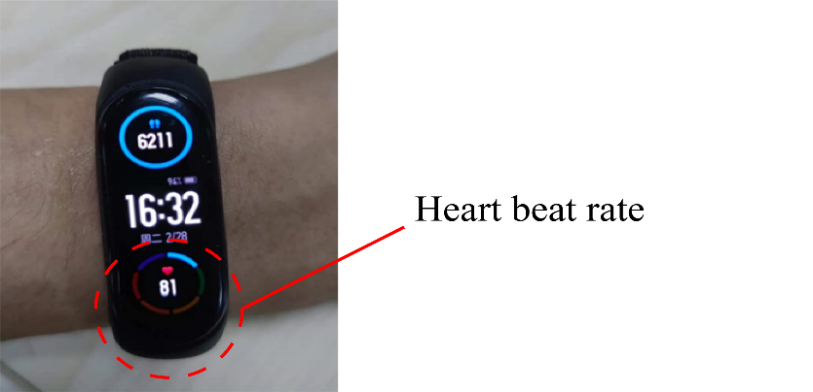


**Figure S5.** Heart rate test by a commercial Mi Band 6


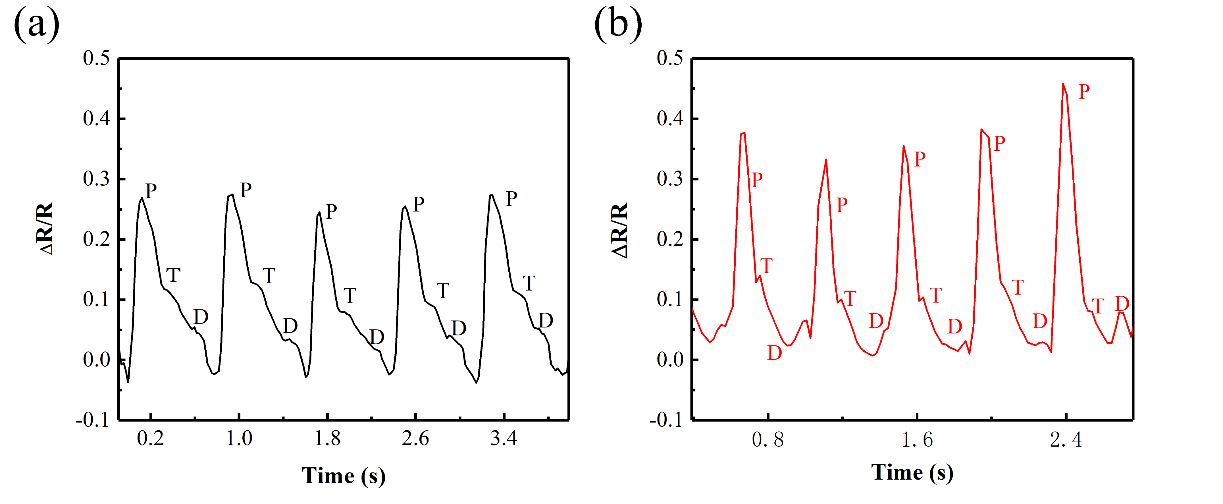


**Figure S6.** The detailed waveform of the radial artery pulse monitoring. (a) Normal relaxed state. (b) After strenuous exercise.


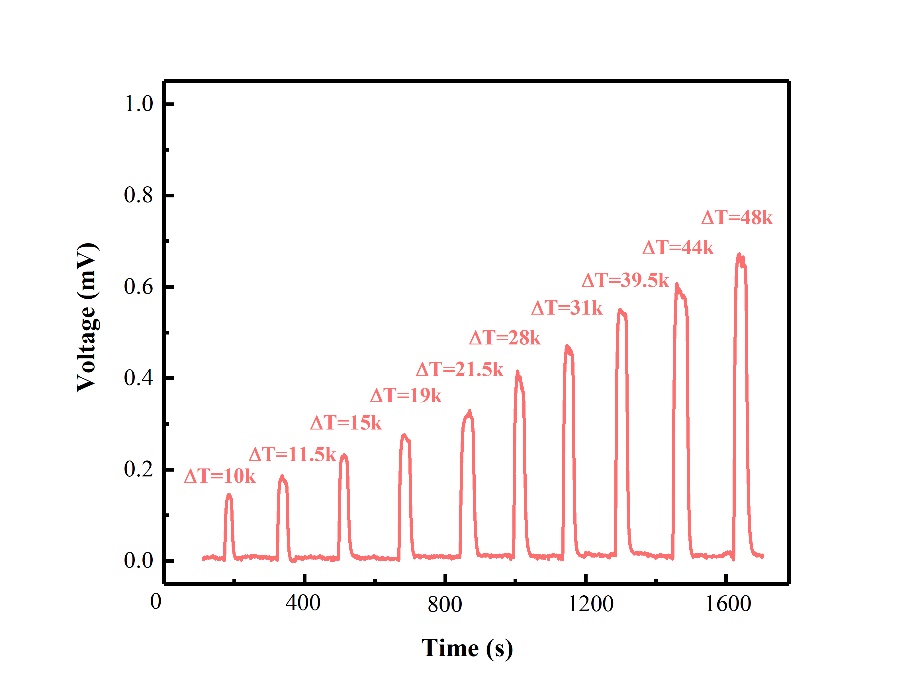


**Figure S7.** Thermal electricity response at different temperature variation.

**Table S1.** The comparation of sensors for pressure sensing

| Materials | Sensing range (kPa) | Sensitivity | Response/recovery time | Stability and repeatability | limit of detection | Reference |
| --- | --- | --- | --- | --- | --- | --- |
| TiO_2_ nanofiber | 0-0.3 | 4.4 | 16 ms | 50000 | 0.8Pa | 1 |
|  | 0.3-13 | 0.073 |  |  |  |  |
|  | 13-110 | 0.015 |  |  |  |  |
| GO-doped PU@PEDOT fibers | 0-1 | 20.6 | 12 ms | 10000 | 1 Pa | 2 |
|  | 1-8 | 0.89 |  |  |  |  |
|  | 8-20 | 0.15 |  |  |  |  |
| Stannum (IV)-doped SrTiO3 nanofiber | 0-0.4 | 2.24 | 12ms /32 ms | 1000 | 20 Pa | 3 |
|  | 0.4-3 | 0.016 |  |  |  |  |
| CCNTs/PEDOT@PU/ CCNTs nanofibrous | 0-1 | 5.13 | 80 ms/ 120 ms | 1000 | 1 Pa | 4 |
|  | 1-8 | 0.14 |  |  |  |  |
| TPU/PVDF-HFP nanofibers | 0-1.2 | 0.718 | 86 ms/ 98 ms | 1000 | 7 Pa | 5 |
|  | 1.2-10 | 0.094 |  |  |  |  |
| Pt/TPU fibers | 0-200 | 0.045 | 125 ms /16 ms | 1000 | / | 6 |
| PVDF nanofiber/PVA-CNTs | 0-40 | 0.0196 | / | 350 | / | 7 |
| fluffy PVDF nanofibers | 0-1 | 1.12 | 10 ms | 10000 | / | 8 |
| Cr/Au/PDMS | 80 | 0.0202 | 100 ms/200 ms | 10000 | / | 9 |
| PANI/CNT/PDMS | 0-130 | 0.918 | 44 ms/33 ms | 10000 | / | 10 |
|  | 130-170 | 0.071 |  |  |  |  |
| Graphene PUA | 6 | 1.63 | <3ms | 10000 | 4.8 Pa | 11 |
|  | 6-100 | 0.04 |  |  |  |  |
| PDMS | 10 | 0.815 | 38 ms | / | 17.5 Pa | 12 |
|  | >150 | 0.0047 |  |  |  |  |
| PAN/Al_2_O_3_ | 4.5 | 1.41 | <300 ms | 5000 | / | 13 |
| PEDOT/MWCNT@PU fibers | 0-8 | 0.12 | 80 ms/ 95 ms | 18000 | 4 Pa | This work |
|  | 8-28 | 1.6 |  |  |  |  |
|  | 28-50 | 0.51 |  |  |  |  |
|  | 50-70 | 0.056 |  |  |  |  |

**Table S2**. Comparison of pressure and temperature sensors of reported studies

| Material | Mechanism | Sensitivity/range | Response/recovery time | Repeatability | Reference |
| --- | --- | --- | --- | --- | --- |
| Cr/Au/PDMS | piezoresistivity | 0.0202 kPa^-1^/80 kPa | 100 ms/200 ms | 10000 | 14 |
|  | piezoresistivity | 0.083 % °C^-1^/60 °C | 14 s | - |  |
| Cotton/rGO/CNT | capacitance | 1.042 kPa^-1^/16 kPa | 50 ms /50 ms | 5000 | 15 |
|  | piezoresistivity | 4.597 %°C^-1^/28-40 °C | 20 s/70 s | 10 |  |
| Silk/AgNW/CNT/ ionic liquid | capacitance | 0.136 kPa^-1^/10 kPa | 250 ms | 5000 | 16 |
|  | piezoresistivity | 1.23 %°C^-1^/65 °C | 16.5 s | 400 |  |
| PANI/CNT/PDMS | piezoresistivity | 0.92 kPa^-1^/130 kPa | 44 ms/33 ms | 10000 | 17 |
|  | thermoelectricity | 17.1 μV°C^-1^/100 k | - | 50 |  |
| PEDOT/WMCNT@PU | piezoresistivity | 1.6 kPa^-1^/70 kPa | 80 ms/ 95 ms | 18000 | This work |
|  | thermoelectricity | 13.2 μV°k^-1^/50 k | 8.7 s/14.8 s | 80 |  |

**Reference**

1. Fu, M.; Zhang, J.; Jin, Y.; Zhao, Y.; Huang, S.; Guo, C. F., A Highly Sensitive, Reliable, and High-Temperature-Resistant Flexible Pressure Sensor Based on Ceramic Nanofibers. *Adv Sci* **2020,** *7* (17), 2000258.

2. Qi, K.; He, J.; Wang, H.; Zhou, Y.; You, X.; Nan, N.; Shao, W.; Wang, L.; Ding, B.; Cui, S., A Highly Stretchable Nanofiber-Based Electronic Skin with Pressure-, Strain-, and Flexion-Sensitive Properties for Health and Motion Monitoring. *ACS Applied Materials & Interfaces* **2017,** *9* (49), 42951-42960.

3. Gao, X.; Zhou, F.; Li, M.; Wang, X.; Chen, S.; Yu, J., Flexible Stannum-Doped SrTiO(3) Nanofiber Membranes for Highly Sensitive and Reliable Piezoresistive Pressure Sensors. *ACS Appl Mater Interfaces* **2021,** 13(44), 52811-52821,.

4. Wang, M.; Dong, L.; Wu, J.; Shi, J.; Gao, Q.; Zhu, C.; Morikawa, H., Leaf-meridian bio-inspired nanofibrous electronics with uniform distributed microgrid and 3D multi-level structure for wearable applications. *npj Flexible Electronics* **2022,** *6* (1).

5. Uzabakiriho, P. C.; Wang, M.; Ma, C.; Zhao, G., Stretchable, breathable, and highly sensitive capacitive and self-powered electronic skin based on core–shell nanofibers. *Nanoscale* **2022,** *14* (17), 6600-6611.

6. Li, Y.; Jia, J.; Yu, H.; Wang, S.; Jin, Z.-Y.; Zhang, Y.-H.; Ma, H.-Z.; Zhang, K.; Ke, K.; Yin, B.; Yang, M.-B., Macromolecule Relaxation Directed 3D Nanofiber Architecture in Stretchable Fibrous Mats for Wearable Multifunctional Sensors. *ACS Applied Materials & Interfaces* **2022,** *14* (13), 15678-15686.

7. Zhou, Q.; Chen, T.; Cao, S.; Xia, X.; Bi, Y.; Xiao, X., A novel flexible piezoresistive pressure sensor based on PVDF/PVA-CNTs electrospun composite film. *Applied Physics A* **2021,** *127* (9).

8. Jin, T.; Pan, Y.; Jeon, G. J.; Yeom, H. I.; Zhang, S.; Paik, K. W.; Park, S. K., Ultrathin Nanofibrous Membranes Containing Insulating Microbeads for Highly Sensitive Flexible Pressure Sensors. *ACS Appl Mater Interfaces* **2020,** *12* (11), 13348-13359.

9. Cai, M.; Jiao, Z.; Nie, S.; Wang, C.; Zou, J.; Song, J., A multifunctional electronic skin based on patterned metal films for tactile sensing with a broad linear response range. Science Advances 2021, 7 (52), eabl8313.

10. Wang, Y.; Mao, H.; Wang, Y.; Zhu, P.; Liu, C.; Deng, Y., 3D geometrically structured PANI/CNT-decorated polydimethylsiloxane active pressure and temperature dual-parameter sensors for man–machine interaction applications. Journal of Materials Chemistry A 2020, 8 (30), 15167-15176

11. Chun S, Son W, Choi C, et al. Bioinspired hairy skin electronics for detecting the direction and incident angle of airflow[J]. ACS applied materials & interfaces, 2019, 11(14): 13608-13615.

12. Li T, Luo H, Qin L, et al. Flexible capacitive tactile sensor based on micropatterned dielectric layer[J]. Small, 2016, 12(36): 5042-5048.

13. Han Z, Cheng Z, Chen Y, et al. Fabrication of highly pressure-sensitive, hydrophobic, and flexible 3D carbon nanofiber networks by electrospinning for human physiological signal monitoring[J]. Nanoscale, 2019, 11(13): 5942-5950.

14. Cai, M.; Jiao, Z.; Nie, S.; Wang, C.; Zou, J.; Song, J., A multifunctional electronic skin based on patterned metal films for tactile sensing with a broad linear response range. Science Advances 2021, 7 (52), eabl8313.

15. Yin, F.; Guo, Y.; Li, H.; Yue, W.; Zhang, C.; Chen, D.; Geng, W.; Li, Y.; Gao, S.; Shen, G., A waterproof and breathable Cotton/rGO/CNT composite for constructing a layer-by-layer structured multifunctional flexible sensor. Nano Research 2022, 15 (10), 9341-9351

16. Wu, R.; Ma, L.; Hou, C.; Meng, Z.; Guo, W.; Yu, W.; Yu, R.; Hu, F.; Liu, X. Y., Silk Composite Electronic Textile Sensor for High Space Precision 2D Combo Temperature-Pressure Sensing. Small 2019, 15 (31), e1901558.

17. Wang, Y.; Mao, H.; Wang, Y.; Zhu, P.; Liu, C.; Deng, Y., 3D geometrically structured PANI/CNT-decorated polydimethylsiloxane active pressure and temperature dual-parameter sensors for man–machine interaction applications. Journal of Materials Chemistry A 2020, 8 (30), 15167-15176.
